# Tetra­aqua­bis(3,5-di-4-pyridyl-1,2,4-triazolato-κ*N*)cobalt(II) dihydrate

**DOI:** 10.1107/S1600536809011982

**Published:** 2009-04-08

**Authors:** Lin Yi Dong

**Affiliations:** a School of Pharmacy, Tianjin Medical University, Tianjin 300070, People’s Republic of China

## Abstract

The Co^II^ atom in the title compound, [Co(C_12_H_8_N_5_)_2_(H_2_O)_4_]·2H_2_O, lies on a center of inversion and is bonded to two *N*-heterocycles and to four water mol­ecules in a slightly distorted octahedral coordination. The coordinated and lattice water mol­ecules inter­act with the *N*-heterocycles through O—H⋯N hydrogen bonds, generating a three-dimensional supra­molecular architecture.

## Related literature

For magnetic studies of transition metal complexes with 1,2,4-triazole derivatives, see: Haasnoot (2000 ▶). For the potential applications of complexes containing substituted 1,2,4-triazole ligands with spin-crossover properties in mol­ecular-based memory devices, displays and optical switches, see: Kahn & Martinez (1998 ▶). For 3,5-di(4-pyridine)-1,2,4-triazole, see: Zhang *et al.* (2006 ▶); Sreenivasulu & Vittal (2004 ▶). For the structure of water, see: Tajkhorshid *et al.* (2002 ▶). For the synthesis, see: Basu & Dutta (1964 ▶). For a trinuclear water cluster, see: König (1944 ▶).
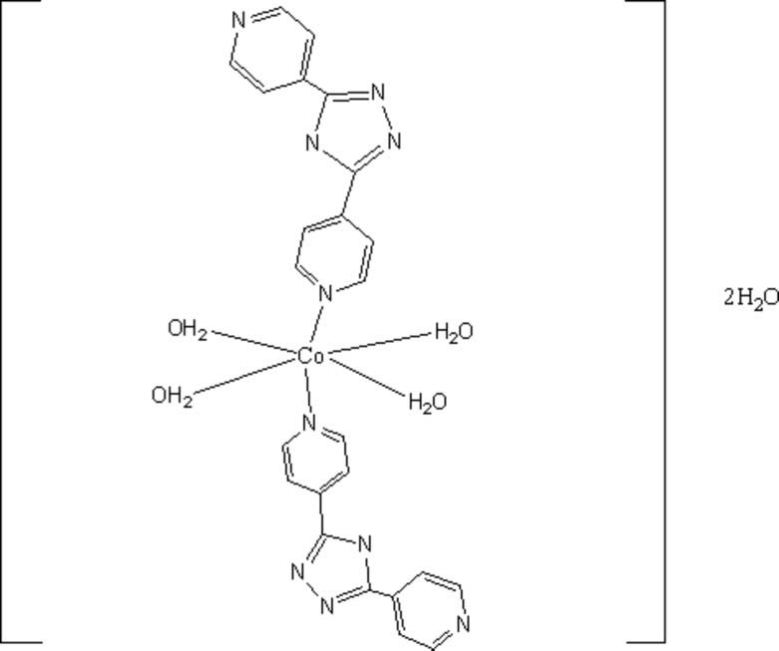


## Experimental

### 

#### Crystal data

[Co(C_12_H_8_N_5_)_2_(H_2_O)_4_]·2H_2_O
*M* *_r_* = 611.49
Monoclinic, 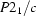

*a* = 7.3660 (15) Å
*b* = 15.654 (3) Å
*c* = 11.857 (2) Å
β = 107.34 (3)°
*V* = 1305.1 (5) Å^3^
*Z* = 2
Mo *K*α radiation
μ = 0.72 mm^−1^
*T* = 293 K
0.40 × 0.20 × 0.12 mm

#### Data collection

Bruker SMART CCD area-detector diffractometer
Absorption correction: multi-scan (*SADABS*; Sheldrick, 1996 ▶) *T*_min_ = 0.842, *T*_max_ = 0.917
11054 measured reflections
2423 independent reflections
2009 reflections with *I* > 2σ(*I*)
*R*_int_ = 0.065

#### Refinement

*R*[*F* ^2^ > 2σ(*F* ^2^)] = 0.054
*wR*(*F* ^2^) = 0.108
*S* = 1.07
2420 reflections
243 parameters
H atoms treated by a mixture of independent and constrained refinement
Δρ_max_ = 0.29 e Å^−3^
Δρ_min_ = −0.41 e Å^−3^

### 

Data collection: *SMART* (Bruker, 1997 ▶); cell refinement: *SAINT* (Bruker, 1997 ▶); data reduction: *SAINT*; program(s) used to solve structure: *SHELXL97* (Sheldrick, 2008 ▶); program(s) used to refine structure: *SHELXL97* (Sheldrick, 2008 ▶); molecular graphics: *DIAMOND* (Brandenburg, 1999 ▶); software used to prepare material for publication: *publCIF* (Westrip, 2009 ▶).

## Supplementary Material

Crystal structure: contains datablocks global, I. DOI: 10.1107/S1600536809011982/ng2566sup1.cif

Structure factors: contains datablocks I. DOI: 10.1107/S1600536809011982/ng2566Isup2.hkl

Additional supplementary materials:  crystallographic information; 3D view; checkCIF report

## Figures and Tables

**Table 1:** Selected geometric parameters (Å, °)

Co1—O1	2.100 (2)
Co1—O2	2.126 (2)
Co1—N1	2.134 (3)

**Table 2:** Hydrogen-bond geometry (Å, °)

*D*—H⋯*A*	*D*—H	H⋯*A*	*D*⋯*A*	*D*—H⋯*A*
O1—H1*A*⋯N3^ii^	0.851 (10)	2.42 (3)	3.070 (4)	134 (3)
O1—H1*A*⋯N4^ii^	0.851 (10)	1.966 (12)	2.803 (4)	167 (4)
O1—H1*B*⋯O3^iii^	0.852 (10)	1.99 (2)	2.791 (4)	155 (3)
O2—H2*A*⋯O3	0.851 (10)	1.973 (14)	2.801 (4)	164 (3)
O2—H2*B*⋯N3^iv^	0.850 (10)	1.973 (19)	2.792 (4)	161 (5)
O2—H2*B*⋯N4^iv^	0.850 (10)	2.60 (4)	3.220 (4)	130 (4)
O3—H3*A*⋯N2^v^	0.852 (10)	2.077 (12)	2.926 (4)	174 (4)
O3—H3*B*⋯N5^vi^	0.849 (10)	1.950 (14)	2.786 (4)	168 (5)

Symmetry codes: (ii) 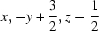; (iii) 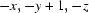; (iv) 
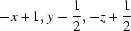
; (v) 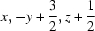; (vi) 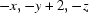.

**Fig. 1.**
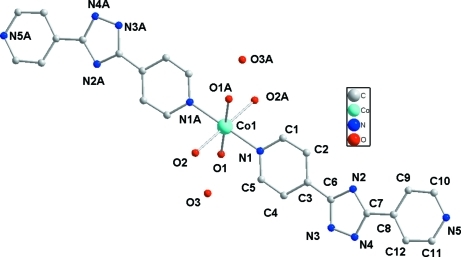
The molecular structure and atom-labeling scheme of (I).

**Fig. 2.**
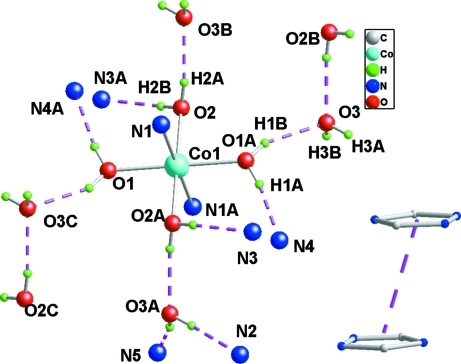
The trinuclear water clusters stabling the packing structure of 1.

## supplementary crystallographic information

### Comment

Transition metal complexes with 1,2,4-triazole derivatives as ligands are of great interest as they are the subject of magnetic studies (Haasnoot, 2000). Some complexes containing substituted 1,2,4-triazole ligands have spin-crossover properties, which could be used in molecular-based memory devices, displays and optical switches (Kahn & Martinez, 1998). The ligand 3,5-di(4-pyridine)-1,2,4-triazole (*L*) is of special interest as it contains multi-dentate donor atoms and shows diverse coordination modes.. Especially only a few examples about the coordinaiton chemistry of *L* are reported. Some unusual coordination modes of *L* also have been reported forming interesting supramolecular isomerism systems (Zhang *et al.*, 2006). On the other hand, water is quite important for our common life (Tajkhorshid *et al.*, 2002). It has been the focus of intense research interests for their unusual properties in biological system and also plays an important role in biological self-assembly processes (Sreenivasulu & Vittal, 2004).

In this work, we synthesized a new compound [Co(*L*)_2_(H_2_O)_4_](H_2_O)_2_ (I) (*L* = 3,5-di(4-pyridine)-1,2,4-triazole). 1 is composed of one cobalt(II) cation, two *L* ligand, four coordinated and two lattice water molecules. The cobalt(II) cation is six-coordinated in the octahedral geometry. The equatorial site of Cobalt cation is occupied by four aqua molecules while the axial site is occupied by two nitrogen atoms of two mono-dentate *L* ligands. The mono-dentate coordination mode of *L* is different from previously reported di-, tri- or tetra-dentate coordination modes of *L*.

O1, O2 from coordination water molecules and O3 from lattice water molecules generate strong intermolecular hydrogen bondings and construct trinuclear water clusters, in which O3 acts as the hydrogen acceptors and O1, O2 act as hydrogen bonding donors. The hydrogen-bonding distances are 2.791 (4) Å (O1—H1B···O3) and 2.801 (4) Å(O2—H2A···O3), respectively. The average O···O distance is 2.796 (4) Å, which is similar to that(2.75 Å) in the structure of ice (König, 1944).

strong N—H···O hydrogen bonds generated from water molecules and nitrogen atoms of pyridine or triazole groups are also observed rusulting in the three-dimensional supramolecular network(Table 2). π-π stacking interactions between two neighboring triazole groups further consolidating the architecture centroid-centroid distance 3.677 (4) Å]

Perspective drawing with the atomic numbering scheme is illustrated in figure 1. Selected geometric parameters (Å, °) for 1 are listed in table 1. Selected hydrogen-bonding geometric parameters (Å, °) for 1 are listed in table 2. The trinuclear water clusters, corresponding N—H···O hydrogen bonds and π-π stacking are shown in figure 2. The three-dimensional supramolecular packing architecture of (I) is shown in figure 3.

### Experimental

The ligand was prepared according to the previous literature (Basu & Dutta, (1964)). [Co(*L*)_2_(H_2_O)_4_](H_2_O) (1) (*L* = 3,5-di(4-pyridine)-1,2,4-triazole) was prepared under the hydrotheraml conditions. [Co(ClO_4_)_2_].6H_2_O (0.2 mmol), *L* (0.2 mmol) and 18 ml water was added to a 25 ml reaction vessel. the reaction vessel was then sealed and subsequently placed in an oven for 140 h at 160°C. well shaped red block crystals were obtained and washed with ethanol.

### Refinement

The carbon-bound H atoms were positioned geometrically and were allowed to ride on their parent C atoms. The water H atoms were located from a difference density map and were refined with distance restraints of O—H = 0.85±0.01 Å.

### Crystal data

**Table:**

[Co(C_12_H_8_N_5_)_2_(H_2_O)_4_]·2(H_2_O)	*F*(000) = 634
*M**_r_* = 611.49	*D*_x_ = 1.556 Mg m^−^^3^
Monoclinic, *P*2_1_/*c*	Mo *K*α radiation, λ = 0.71073 Å
Hall symbol: -P 2ybc	Cell parameters from 556 reflections
*a* = 7.3660 (15) Å	θ = 1.5–25.5°
*b* = 15.654 (3) Å	µ = 0.72 mm^−^^1^
*c* = 11.857 (2) Å	*T* = 293 K
β = 107.34 (3)°	Block, red
*V* = 1305.1 (5) Å^3^	0.40 × 0.20 × 0.12 mm
*Z* = 2	

### Data collection

**Table:**

Bruker SMART CCD area-detector diffractometer	2423 independent reflections
Radiation source: fine-focus sealed tube	2009 reflections with *I* > 2σ(*I*)
graphite	*R*_int_ = 0.065
φ and ω scans	θ_max_ = 25.5°, θ_min_ = 3.2°
Absorption correction: multi-scan (*SADABS*; Sheldrick, 1996)	*h* = −8→8
*T*_min_ = 0.842, *T*_max_ = 0.917	*k* = −18→18
11054 measured reflections	*l* = −14→14

### Refinement

**Table:**

Refinement on *F*^2^	0 restraints
Least-squares matrix: full	H atoms treated by a mixture of independent and constrained refinement
*R*[*F*^2^ > 2σ(*F*^2^)] = 0.054	*w* = 1/[σ^2^(*F*_o_^2^) + (0.0298*P*)^2^ + 0.2298*P*] where *P* = (*F*_o_^2^ + 2*F*_c_^2^)/3
*wR*(*F*^2^) = 0.108	(Δ/σ)_max_ < 0.001
*S* = 1.07	Δρ_max_ = 0.29 e Å^−^^3^
2420 reflections	Δρ_min_ = −0.41 e Å^−^^3^
243 parameters	

### Special details

**Table:**

Geometry. All e.s.d.'s (except the e.s.d. in the dihedral angle between two l.s. planes) are estimated using the full covariance matrix. The cell e.s.d.'s are taken into account individually in the estimation of e.s.d.'s in distances, angles and torsion angles; correlations between e.s.d.'s in cell parameters are only used when they are defined by crystal symmetry. An approximate (isotropic) treatment of cell e.s.d.'s is used for estimating e.s.d.'s involving l.s. planes.
Refinement. Refinement of *F*^2^ against ALL reflections. The weighted *R*-factor *wR* and goodness of fit *S* are based on *F*^2^, conventional *R*-factors *R* are based on *F*, with *F* set to zero for negative *F*^2^. The threshold expression of *F*^2^ > σ(*F*^2^) is used only for calculating *R*-factors(gt) *etc*. and is not relevant to the choice of reflections for refinement. *R*-factors based on *F*^2^ are statistically about twice as large as those based on *F*, and *R*- factors based on ALL data will be even larger.

### Fractional atomic coordinates and isotropic or equivalent isotropic displacement parameters (Å^2^)

**Table:**

	*x*	*y*	*z*	*U*_iso_*/*U*_eq_	
C1	0.3827 (5)	0.6826 (2)	−0.0776 (3)	0.0306 (8)	
C2	0.3536 (5)	0.7690 (2)	−0.0713 (3)	0.0299 (8)	
C3	0.3919 (4)	0.80864 (19)	0.0377 (3)	0.0218 (7)	
C4	0.4634 (5)	0.7565 (2)	0.1360 (3)	0.0290 (8)	
C5	0.4886 (5)	0.6711 (2)	0.1224 (3)	0.0294 (8)	
C6	0.3518 (5)	0.8987 (2)	0.0514 (3)	0.0229 (7)	
C7	0.2496 (5)	1.02433 (19)	0.0220 (3)	0.0241 (7)	
C8	0.1690 (5)	1.1049 (2)	−0.0324 (3)	0.0244 (7)	
C9	0.1117 (5)	1.1170 (2)	−0.1528 (3)	0.0330 (9)	
C10	0.0440 (6)	1.1952 (2)	−0.1990 (3)	0.0368 (9)	
C11	0.0861 (6)	1.2510 (3)	−0.0181 (4)	0.0450 (11)	
C12	0.1540 (6)	1.1751 (2)	0.0357 (4)	0.0403 (10)	
Co1	0.5000	0.5000	0.0000	0.02203 (19)	
H1	0.363 (5)	0.657 (2)	−0.147 (3)	0.033 (10)*	
H2	0.313 (5)	0.799 (3)	−0.138 (3)	0.046 (12)*	
H4	0.495 (5)	0.781 (2)	0.209 (3)	0.034 (10)*	
H5	0.532 (5)	0.637 (2)	0.187 (3)	0.036 (10)*	
H9	0.118 (5)	1.075 (2)	−0.199 (3)	0.028 (10)*	
H10	0.013 (6)	1.203 (3)	−0.278 (4)	0.052 (13)*	
H11	0.078 (6)	1.299 (3)	0.026 (4)	0.057 (13)*	
H12	0.188 (6)	1.170 (3)	0.116 (4)	0.051 (12)*	
H1A	0.261 (5)	0.484 (3)	−0.2116 (18)	0.069 (16)*	
H2A	0.276 (4)	0.5057 (16)	0.135 (3)	0.031 (10)*	
H3A	0.163 (5)	0.571 (2)	0.2816 (10)	0.041 (12)*	
H1B	0.143 (3)	0.475 (3)	−0.140 (3)	0.077 (17)*	
H2B	0.416 (6)	0.446 (3)	0.1861 (19)	0.084 (18)*	
H3B	0.081 (6)	0.6269 (11)	0.194 (4)	0.069 (16)*	
N1	0.4483 (4)	0.63277 (16)	0.0175 (2)	0.0262 (6)	
N2	0.2624 (4)	0.95193 (16)	−0.0378 (2)	0.0229 (6)	
N3	0.3929 (4)	0.93481 (17)	0.1575 (2)	0.0295 (7)	
N4	0.3253 (4)	1.01645 (17)	0.1389 (2)	0.0308 (7)	
N5	0.0298 (4)	1.26290 (18)	−0.1346 (3)	0.0371 (8)	
O1	0.2504 (3)	0.49368 (17)	−0.1431 (2)	0.0334 (6)	
O2	0.3487 (4)	0.46767 (16)	0.1212 (2)	0.0302 (6)	
O3	0.1157 (4)	0.57516 (16)	0.2069 (2)	0.0329 (6)	

### Atomic displacement parameters (Å^2^)

**Table:**

	*U*^11^	*U*^22^	*U*^33^	*U*^12^	*U*^13^	*U*^23^
C1	0.047 (2)	0.0220 (18)	0.0201 (18)	0.0038 (16)	0.0054 (16)	−0.0033 (15)
C2	0.044 (2)	0.0184 (17)	0.0245 (19)	0.0057 (15)	0.0057 (16)	0.0020 (15)
C3	0.0241 (18)	0.0184 (16)	0.0231 (17)	0.0017 (13)	0.0074 (14)	−0.0003 (13)
C4	0.043 (2)	0.0239 (18)	0.0188 (18)	0.0052 (16)	0.0075 (16)	−0.0032 (15)
C5	0.043 (2)	0.0200 (18)	0.0232 (19)	0.0050 (15)	0.0065 (16)	0.0043 (14)
C6	0.0277 (18)	0.0182 (16)	0.0224 (17)	−0.0006 (14)	0.0066 (14)	0.0000 (13)
C7	0.0297 (18)	0.0194 (17)	0.0227 (17)	−0.0001 (13)	0.0072 (14)	0.0002 (13)
C8	0.0247 (18)	0.0211 (17)	0.0269 (18)	−0.0023 (14)	0.0067 (14)	−0.0009 (14)
C9	0.044 (2)	0.0231 (19)	0.028 (2)	0.0046 (16)	0.0043 (17)	−0.0031 (16)
C10	0.044 (2)	0.033 (2)	0.027 (2)	0.0034 (17)	0.0023 (18)	0.0073 (17)
C11	0.067 (3)	0.024 (2)	0.045 (3)	0.013 (2)	0.017 (2)	−0.0019 (18)
C12	0.065 (3)	0.027 (2)	0.028 (2)	0.0129 (18)	0.011 (2)	0.0002 (16)
Co1	0.0282 (4)	0.0162 (3)	0.0206 (3)	0.0025 (3)	0.0055 (2)	0.0004 (3)
N1	0.0358 (17)	0.0170 (14)	0.0251 (15)	0.0032 (12)	0.0081 (12)	−0.0001 (11)
N2	0.0283 (15)	0.0154 (14)	0.0241 (15)	0.0011 (11)	0.0063 (12)	−0.0011 (11)
N3	0.0398 (18)	0.0213 (15)	0.0255 (16)	0.0099 (13)	0.0065 (13)	0.0017 (12)
N4	0.0461 (18)	0.0213 (16)	0.0221 (15)	0.0069 (13)	0.0057 (13)	−0.0013 (11)
N5	0.044 (2)	0.0223 (16)	0.044 (2)	0.0079 (14)	0.0115 (16)	0.0055 (14)
O1	0.0312 (14)	0.0408 (15)	0.0264 (14)	−0.0029 (13)	0.0058 (10)	−0.0009 (12)
O2	0.0346 (15)	0.0295 (13)	0.0296 (14)	0.0062 (11)	0.0142 (12)	0.0021 (11)
O3	0.0436 (16)	0.0239 (14)	0.0286 (15)	0.0017 (12)	0.0067 (12)	0.0018 (11)

### Geometric parameters (Å, °)

**Table:**

C1—N1	1.337 (4)	C10—N5	1.328 (5)
C1—C2	1.376 (5)	C10—H10	0.90 (4)
C1—H1	0.89 (4)	C11—N5	1.331 (5)
C2—C3	1.385 (5)	C11—C12	1.370 (5)
C2—H2	0.89 (4)	C11—H11	0.93 (4)
C3—C4	1.392 (5)	C12—H12	0.92 (4)
C3—C6	1.459 (4)	Co1—O1^i^	2.100 (2)
C4—C5	1.365 (5)	Co1—O1	2.100 (2)
C4—H4	0.91 (4)	Co1—O2^i^	2.126 (2)
C5—N1	1.332 (4)	Co1—O2	2.126 (2)
C5—H5	0.91 (4)	Co1—N1^i^	2.134 (3)
C6—N3	1.329 (4)	Co1—N1	2.134 (3)
C6—N2	1.354 (4)	N3—N4	1.365 (4)
C7—N4	1.336 (4)	O1—H1A	0.851 (10)
C7—N2	1.355 (4)	O1—H1B	0.852 (10)
C7—C8	1.459 (4)	O2—H2A	0.851 (10)
C8—C9	1.377 (5)	O2—H2B	0.850 (10)
C8—C12	1.387 (5)	O3—H3A	0.852 (10)
C9—C10	1.373 (5)	O3—H3B	0.849 (10)
C9—H9	0.87 (4)		
			
N1—C1—C2	123.4 (3)	C11—C12—C8	119.8 (4)
N1—C1—H1	116 (2)	C11—C12—H12	121 (3)
C2—C1—H1	121 (2)	C8—C12—H12	120 (3)
C1—C2—C3	120.0 (3)	O1^i^—Co1—O1	180.0
C1—C2—H2	119 (3)	O1^i^—Co1—O2^i^	91.47 (10)
C3—C2—H2	121 (3)	O1—Co1—O2^i^	88.53 (10)
C2—C3—C4	116.1 (3)	O1^i^—Co1—O2	88.53 (10)
C2—C3—C6	123.0 (3)	O1—Co1—O2	91.47 (10)
C4—C3—C6	120.8 (3)	O2^i^—Co1—O2	180.0
C5—C4—C3	120.4 (3)	O1^i^—Co1—N1^i^	89.19 (10)
C5—C4—H4	122 (2)	O1—Co1—N1^i^	90.81 (10)
C3—C4—H4	118 (2)	O2^i^—Co1—N1^i^	91.23 (10)
N1—C5—C4	123.5 (3)	O2—Co1—N1^i^	88.77 (10)
N1—C5—H5	116 (2)	O1^i^—Co1—N1	90.81 (10)
C4—C5—H5	120 (2)	O1—Co1—N1	89.19 (10)
N3—C6—N2	113.4 (3)	O2^i^—Co1—N1	88.77 (10)
N3—C6—C3	121.3 (3)	O2—Co1—N1	91.23 (10)
N2—C6—C3	125.1 (3)	N1^i^—Co1—N1	180.0
N4—C7—N2	113.1 (3)	C5—N1—C1	116.7 (3)
N4—C7—C8	121.8 (3)	C5—N1—Co1	122.2 (2)
N2—C7—C8	125.0 (3)	C1—N1—Co1	121.0 (2)
C9—C8—C12	116.1 (3)	C6—N2—C7	101.5 (3)
C9—C8—C7	122.5 (3)	C6—N3—N4	106.0 (2)
C12—C8—C7	121.3 (3)	C7—N4—N3	105.8 (2)
C10—C9—C8	120.0 (3)	C10—N5—C11	115.6 (3)
C10—C9—H9	120 (2)	Co1—O1—H1A	118 (3)
C8—C9—H9	120 (2)	Co1—O1—H1B	126 (3)
N5—C10—C9	124.3 (4)	H1A—O1—H1B	109.1 (17)
N5—C10—H10	117 (3)	Co1—O2—H2A	117 (2)
C9—C10—H10	119 (3)	Co1—O2—H2B	115 (3)
N5—C11—C12	124.2 (4)	H2A—O2—H2B	109.4 (15)
N5—C11—H11	115 (3)	H3A—O3—H3B	106 (4)
C12—C11—H11	121 (3)		
			
N1—C1—C2—C3	−0.3 (6)	C2—C1—N1—Co1	−177.5 (3)
C1—C2—C3—C4	1.4 (5)	O1^i^—Co1—N1—C5	−36.4 (3)
C1—C2—C3—C6	−175.5 (3)	O1—Co1—N1—C5	143.6 (3)
C2—C3—C4—C5	−1.3 (5)	O2^i^—Co1—N1—C5	−127.9 (3)
C6—C3—C4—C5	175.6 (3)	O2—Co1—N1—C5	52.1 (3)
C3—C4—C5—N1	0.1 (6)	N1^i^—Co1—N1—C5	−122 (27)
C2—C3—C6—N3	−179.1 (3)	O1^i^—Co1—N1—C1	140.0 (3)
C4—C3—C6—N3	4.2 (5)	O1—Co1—N1—C1	−40.0 (3)
C2—C3—C6—N2	4.7 (5)	O2^i^—Co1—N1—C1	48.5 (3)
C4—C3—C6—N2	−172.0 (3)	O2—Co1—N1—C1	−131.5 (3)
N4—C7—C8—C9	171.9 (3)	N1^i^—Co1—N1—C1	55 (27)
N2—C7—C8—C9	−5.8 (5)	N3—C6—N2—C7	−0.6 (4)
N4—C7—C8—C12	−5.0 (5)	C3—C6—N2—C7	175.9 (3)
N2—C7—C8—C12	177.3 (3)	N4—C7—N2—C6	0.0 (4)
C12—C8—C9—C10	−0.2 (6)	C8—C7—N2—C6	177.9 (3)
C7—C8—C9—C10	−177.4 (3)	N2—C6—N3—N4	0.8 (4)
C8—C9—C10—N5	0.5 (6)	C3—C6—N3—N4	−175.7 (3)
N5—C11—C12—C8	0.6 (7)	N2—C7—N4—N3	0.5 (4)
C9—C8—C12—C11	−0.3 (6)	C8—C7—N4—N3	−177.5 (3)
C7—C8—C12—C11	176.9 (4)	C6—N3—N4—C7	−0.8 (4)
C4—C5—N1—C1	1.1 (5)	C9—C10—N5—C11	−0.2 (6)
C4—C5—N1—Co1	177.6 (3)	C12—C11—N5—C10	−0.3 (6)
C2—C1—N1—C5	−1.0 (5)		

Symmetry codes: (i) −*x*+1, −*y*+1, −*z*.

### Hydrogen-bond geometry (Å, °)

**Table:**

*D*—H···*A*	*D*—H	H···*A*	*D*···*A*	*D*—H···*A*
O1—H1A···N3^ii^	0.85 (1)	2.42 (3)	3.070 (4)	134 (3)
O1—H1A···N4^ii^	0.85 (1)	1.97 (1)	2.803 (4)	167 (4)
O1—H1B···O3^iii^	0.85 (1)	1.99 (2)	2.791 (4)	155 (3)
O2—H2A···O3	0.85 (1)	1.97 (1)	2.801 (4)	164 (3)
O2—H2B···N3^iv^	0.85 (1)	1.97 (2)	2.792 (4)	161 (5)
O2—H2B···N4^iv^	0.85 (1)	2.60 (4)	3.220 (4)	130 (4)
O3—H3A···N2^v^	0.85 (1)	2.08 (1)	2.926 (4)	174 (4)
O3—H3B···N5^vi^	0.85 (1)	1.95 (1)	2.786 (4)	168 (5)

Symmetry codes: (ii) *x*, −*y*+3/2, *z*−1/2; (iii) −*x*, −*y*+1, −*z*; (iv) −*x*+1, *y*−1/2, −*z*+1/2; (v) *x*, −*y*+3/2, *z*+1/2; (vi) −*x*, −*y*+2, −*z*.
